# Oxytocin Receptor Polymorphism Decreases Midline Neural Activations to Social Stimuli in Anorexia Nervosa

**DOI:** 10.3389/fpsyg.2018.02183

**Published:** 2018-11-13

**Authors:** Margarita Sala, Kihwan Han, Summer Acevedo, Daniel C. Krawczyk, Carrie J. McAdams

**Affiliations:** ^1^Department of Psychology, Southern Methodist University, Dallas, TX, United States; ^2^Center for Brain Health, University of Texas at Dallas, Dallas, TX, United States; ^3^Department of Psychiatry, University of Texas at Southwestern Medical School, Dallas, TX, United States

**Keywords:** social cognition, fMRI, eating disorders, neuroimaging, self-perception, depression, anxiety, endophenotypes

## Abstract

Oxytocin is a neurotransmitter related to both feeding and social behavior; anorexia nervosa is a psychiatric illness defined by reduced food intake, weight loss, and problems in social perceptions. Oxytocin receptor single nucleotide polymorphisms rs2254298 or rs53576 and neural responses to social stimuli were evaluated in adult women with or recovered from anorexia nervosa using functional magnetic resonance imaging. Carriers of the A allele for *OXTR* rs2254298 (2 AA and 10 AG) showed significantly reduced activation of portions of the posterior cingulate cortex and medial prefrontal cortex for social stimuli as well as greater negative connectivity between the posterior cingulate and the occipital lobe relative to the GG subjects for rs2254298. Differences in the other *OXTR* SNP, rs53576, did not result in detectable neural differences in either whole brain or region of interest analyses. Development of a mechanistic, biological model of how social behavior is impacted by mental illness requires linking genes to functional brain activations in disease. This pilot study suggests that in anorexia nervosa, differences related to *OXTR* SNP rs2254298 may alter neural responses to social stimuli and disrupt the engagement and disengagement of the default mode network.

## Introduction

Anorexia nervosa (AN) is an illness that includes altered perceptions related to body-image and self-esteem in concert with an inability to maintain a healthy body weight. Differences in social behaviors are observed in AN, such as impaired recognition of the emotional content in faces, and reduced mentalization (Oldershaw et al., 2011; Tchanturia et al., 2011; Lang et al., 2016). Neural differences in regions important for social cognitive processing, such as the precuneus, temporoparietal junctions, medial prefrontal cortex, and inferior frontal gyri, have also been reported in this disease (McAdams and Krawczyk, 2011, 2014; Schulte-Ruther et al., 2012; McAdams et al., 2015, 2016), but it remains unclear how genetic differences might impact social brain function in AN.

Starvation is an environmental stressor present in all patients with AN, and it impacts many biological pathways, with effects on both reproduction and metabolism. The oxytocin system promotes social behaviors in mammals, including reproduction, pair-bonding, and maternal-infant attachment (Feldman et al., 2016). The down-regulation of oxytocin systems in response to the environmental stress associated with starvation may be beneficial as social behaviors are disadvantageous under such conditions. In AN, hypermethylation of the oxytocin receptor has been observed (Kim et al., 2014a; Booij et al., 2015), and reduced oxytocin levels were found in cerebral spinal fluid (Frank et al., 2000).

Polymorphisms of the oxytocin receptor gene (*OXTR*) in healthy populations have been related to differences in personality characteristics, including neuroticism, self-esteem, anxiety, and autistic traits (Feldman et al., 2016). Differences in these personality traits have also been observed in AN (Cervera et al., 2003; Godart et al., 2003; Anckarsater et al., 2012). Both restrictive and purging eating behaviors have been associated with oxytocin receptor variants in a community sample, with the GG rs53576 genotype associated with both binge-eating and purging behaviors, and the AG/AA rs2253298 genotype associated with lifetime restrictive eating behaviors (Micali et al., 2017). Administration of oxytocin to patients with AN is now being explored as a potential treatment (Kim et al., 2014b,c, 2015). In healthy populations, brain structure and function have been shown to differ based on both *OXT* and *OXTR* polymorphisms (Inoue et al., 2010; Tost et al., 2010, 2011; Furman et al., 2011; Love et al., 2012; Chang et al., 2014). By examining oxytocin receptor polymorphisms in concert with brain function in women with AN, we evaluate how molecular changes in this pathway impact the neural circuits involved in social perception.

In a pilot study using the Social Attribution Task in 3T fMRI, we found differences in neural responses to social stimuli in 17 women recently diagnosed with AN and 17 healthy control women (McAdams and Krawczyk, 2011). Recently, we found that two polymorphisms of the *OXTR*, rs53576 and rs2254298, were related to the severity of eating disorder symptoms (Acevedo et al., 2015). Both *OXTR* rs53576 (minor allele frequency 0.39; range 0.19 to 0.65) and rs2254298 (minor allele frequency 0.21; range 0.10–0.34) are common intron variants whose frequency varies in different populations although the molecular processes impacted by these minor alleles is unknown (Genomes Project Consortium Auton et al., 2015). This study explores how gene-environment can interact by considering social brain function in the context of a common genetic polymorphism using a disease model defined by the presence of the environmental stressor of starvation. The goal of the study was to assess whether single nucleotide polymorphisms (SNP) of the oxytocin receptor gene (*OXTR*) could be mechanistically related to regional differences in functional neural activity in response to social stimuli in women with AN.

## Methods

### Participants

A total of 49 female participants (age range 18–47 years) were enrolled and participated in the study from 2009 to 2015. All participants were recruited from the Dallas, TX area, and provided written informed consent to participate in three different protocols approved by the UT Southwestern Institutional Review Board. Only participants able to complete in both an imaging study and the genetic study were included. Two protocols with the same neuroimaging task were conducted sequentially in time: study 1, 14 subjects included here that enrolled in 2009–2011 (McAdams and Krawczyk, 2011), and study 2, 35 subjects included here that enrolled 2012–2015 (McAdams et al., 2015); a third protocol involving the collection of the DNA began in 2010 (Acevedo et al., 2015).

All subjects were interviewed at first visit using the Structured Clinical Interview for DSM-IV disorders (SCID-RV) to confirm history of or current symptoms of AN in these participants. For enrollment in the first and second imaging protocols, different clinical criteria were utilized to determine the degree of recovery from AN. Both studies still required subjects to have a stable or increasing weight for at least 2 months prior to the scan to minimize the chances of neural differences caused by acute effects of starvation or dehydration (McAdams and Krawczyk, 2011; McAdams et al., 2015). Demographic information was also obtained at first interview. Diagnoses of anxiety disorders and subjects with a history of major depression were permissible, but subjects currently meeting criteria for major depression as well as those with current or past substance dependence, current or past bipolar disorders, and current or past psychotic disorders were not eligible for imaging studies. Participants were screened for MRI compatibility. Body mass index was measured the day of the MRI scan.

### Assessments

Three clinician-based measures assessed depression, anxiety, and eating disorder symptoms. The Quick Inventory of Depressive Symptomatology (QIDS-CR, Rush et al., 2003) is a 16 question inventory of depressive symptoms. The Structured Interview Guide for the Hamilton Anxiety Scale (SIGH-A, Shear et al., 2001) assessed anxiety symptoms in fourteen categories. The Yale Brown Cornell Eating Disorder Survey (YBC-EDS, Mazure et al., 1994; Sunday et al., 1995) is a self-report checklist followed by a 22-statement clinician-administered ED inventory about eating preoccupations and rituals.

Each participant also completed a self-report packet. The Young-Brown Obsessive-Compulsive Symptoms (Y-BOCS, Woody et al., 1995) provided an overall estimate of obsessions and compulsions, including those unrelated to the ED. The 26-item Eating Attitudes Test (EAT-26, Berland et al., 1986) provided a second measure of ED behaviors with subscales for dieting behavior (EAT-D), bulimia behaviors (EAT-B), and oral control behaviors (EAT-O). The 34-item Body Shape Questionnaire (BSQ, Rosen et al., 1996) provided a measure of ED symptomatology related to shape and weight concerns.

### DNA processing

Blood samples were collected from patients and submitted to McDermott Center for Human Growth & Development Human Genetics Clinical Laboratory, located at UT Southwestern Medical Center in Dallas, TX, for processing and isolation of DNA. Samples were quantified using nanodrop and diluted in 96 well plates. The plated samples were then sent for SNP analysis at McDermott Center for Human Growth & Development DNA Sanger Sequencing Core located at UT Southwestern Medical Center in Dallas, TX. Two oxytocin receptor SNPs were premade and purchased from Applied Biosystems by life technology: rs53576/CG (G/A) and rs2254298 (G/A).

### Functional magnetic resonance imaging (fMRI) task

**Figure 1:**
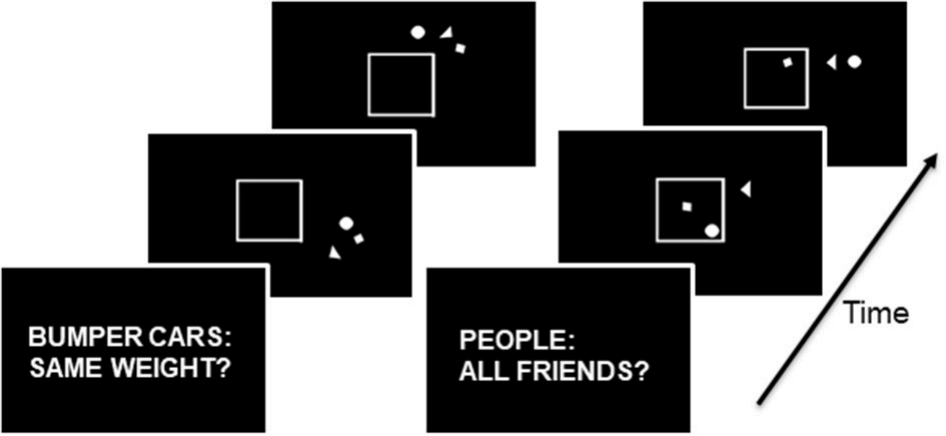
On the left, the progression of stimuli in the Bumper condition: the three shapes move around the middle box with occasional bumps, and the box may open and close during the movements. On the right, the progression of stimuli in the Friends condition: the three shapes move toward and away from each other and in and out of the box which may open and close. During the Bumper condition, the subject engages in visuospatial decision-making. During the Friends condition, the subject engages in social cognitive decision-making.

Subjects viewed videos showing simple shapes moving in two conditions following a 3 s cue screen (Figure 1; Schultz et al., 2003; McAdams and Krawczyk, 2011). In the visuospatial condition, the cue was “Bumper cars: Same weight?” and in the social attribution condition, the cue was “People: All friends?” A total of 16 video clips were presented, eight for each condition. Each video clip presented a circle, a triangle, and a square moving around a white box with one side that could open or close. The shapes moved differently in the two conditions. The small shapes circled the box colliding intermittently in the Bumper videos, whereas they exhibited smaller movements toward and away from one another in the People videos. For the Bumper condition, subjects were instructed to consider whether the interactions between shapes were symmetric, suggesting shapes of equal weight, or asymmetric, suggesting shapes of disparate weight. During the People condition, subjects evaluated whether the interactions of the shapes suggested friendship or not. Subjects provided their response following the video. For each subject, the average reaction time and accuracy of their responses was calculated.

### MRI acquisition

All images were acquired with the same 3T Philips Achieva MRI scanner. Functional images were acquired during 4 runs, each run lasting 128 s, using a 1-shot gradient T2^*^-weighted echoplanar (EPI) image sequence sensitive to blood oxygen level-dependent (BOLD) contrast, a 22.0 cm^2^ field of view, a matrix size of 64x64 and a voxel size of 3.4 × 3.4 × 3 mm. Each sequence was acquired using a repetition time (TR) of 1.5 s, an echo time (TE) of 25 ms, and a flip angle of 60°. Volumes were composed of 33 tilted axial slices (3 mm thick, 1 mm gap). Head motion was limited using foam padding. High resolution MP-RAGE 3D T1-weighted images were acquired for anatomical localization with the following imaging parameters: TR = 2100 ms, TE = 3.7 ms; slice thickness of 1 mm with no gap, a 12° flip angle, and 1 mm^3^ voxels.

### fMRI task activations

Prior to statistical analyses, preprocessing consisted of spatial realignment to the first volume of acquisition, normalization to the MNI standard template, and spatial smoothing with a 6 mm 3D Gaussian kernel. Functional MRI task data were analyzed using Statistical Parametric Mapping software (SPM) run in Matlab 2012 and viewed in both SPM and xjView. An event-related design examined the BOLD signal during the 17 s presentation of each video (events: *People* and *Bumper*). A general linear model created contrast images for each event. Evoked activation was assessed using multiple regression analysis set as boxcar functions. Each regressor was convolved with a canonical hemodynamic response function (HRF) provided in SPM and entered into the modified general linear model (GLM). Parameter estimates (i.e., β values) were extracted from this GLM analysis for each regressor. Resulting single-subject one-sample *t*-test contrast images were examined, and combined to create a group map. The People—Bumper contrast identified neural regions important for social cognitive processing and the Bumper—People contrast identified regions engaged more for the visuospatial analyses (threshold: voxel-wise *p* < 0.005, uncorrected followed by cluster-correction to *p*_*FWE*_ = 0.05). Each of these areas became a region of interest (ROI) for subsequent analyses.

### fMRI connectivity analyses

Task-related functional connectivity was identified using the generalized psychophysiological interaction toolbox (McLaren et al., 2012). Based on the patterns of group differences in BOLD activity under the People—Bumper contrast (see section Results), we examined seed-based functional connectivity under the People—Bumper contrast by seeding at the posterior cingulate cortex (−8, −56, 26) and medial prefrontal cortex (−6, 52, −2) with 6 mm radius spheres. Seed-based functional connectivity examines how strongly the activity in other voxels in the brain are correlated with activity at that defined seed. These seed locations were selected based on the resting-state literature on the default mode network (Andrews-Hanna et al., 2010). For connectivity analysis, the GLM was revised to additionally include psychophysiological terms for the People and Bumper conditions, respectively. In this revised GLM, we obtained estimated beta values for the People and Bumper conditions for each of the subjects. Subsequently, we performed a two-sample *t*-test on the People—Bumper contrast at each of the voxels for group comparisons (*p*_voxel_ < 0.005, *p*_FWE_ < 0.05).

### Genetic analysis of neural data

Whole-brain, two-sample *t*-tests were conducted in SPM 8, in which the subjects were divided by genotype for the dominant *OXTR* polymorphisms (threshold: voxel-wise *p* < 0.005, with cluster-correction to *p*_*FWE*_ < 0.05). To complement the whole-brain analysis, parameter estimates were extracted for each subject for individual ROIs from the People—Bumper and Bumper—People contrasts, and exported to SPSS for subsequent *t*-tests. These *t*-tests were exploratory, using a standard *p* < 0.05, without correction for multiple comparisons; effect sizes of any observed differences are reported, as the primary goal is to explore relationships between genetic polymorphisms and brain function.

### Group comparisons

Student's *t*-tests were used to compare demographic, clinical, and behavioral data, and the beta values were extracted from the ROIs for the two polymorphisms of rs2254298 and rs53576. These analyses were conducted in SPSS software (version 21). Multivariate linear regressions assessed whether individual participants' clinical symptom scores, including the QIDS, SIGHA, EAT, BSQ, and BMI, were related to the neural activations within each of the four ROIs, with Bonferroni correction for multiple comparisons (criterion: *p* < 0.05/number of ROIs).

To further consider the effects of weight-restoration on neural function in these regions, the participants were also divided into groups of weight-restored (BMI > 19, *n* = 25, 6 rs2254298A, 19 rs2254298GG) and underweight (BMI < 19, *n* = 24, 6 rs2254298A, 18 rs2254298GG). Beta values were extracted for each subject for each of the ROIs with significant differences based on SNP of rs2254298. Analysis of variance was used to determine if effects of weight, genotype, or interactions of these variables could be detected, using Bonferroni correction for multiple comparisons (criterion: *p* < 0.05/ the number of ROIs).

## Results

### Demographic, clinical, and behavioral differences in *OXTR* SNP

For rs2254298, there were 12 women who were A carriers (2 AA and 10 AG) and 37 women were GG carriers; this disparate sample size is a limitation of the work. Consistent with our earlier and larger genetic study (Acevedo et al., 2015), the A carriers of rs2254298 showed elevated scores for the Eating Attitudes Test, including the overall EAT, and the subscales for dieting and bulimia (Table 1). No other differences in demographic characteristics of the groups or clinical measures were observed. For rs53576, 27 women were A carriers (5 AA and 21 AG) and 22 women were GG carriers. There were no significant differences related to demographic or clinical assessments when considering rs53576 alone (Supplemental Table 1). This difference from our prior publication may be attributed to the inclusion of 7 individuals that were A carriers for rs2254298 but GG for rs53576 in the GG group, as well as the additional subjects (women with AN that did not participate in the MRI studies) and groups (women with bulimia nervosa and healthy women). No behavioral differences in reaction times or the mean percentage of trials correct responses for either the bumper or the people conditions were observed for the A carriers vs. the GG subjects for either the rs2254298 genotype or the rs53567 genotype using *t*-tests (criterion of *P* < 0.05; Supplemental Table 2).

**Table 1:** Demographic and clinical characteristics for OXTR SNP rs2254298.

	**rs2254298** ***(M, SD)***		**rs2254298 GG vs. A carrier**	
	**GG (*n* = 37)**	**GA/AA (*n* = 12)**	***T***	***p***
Age	26.22 (8.13)	29.67 (7.62)	−1.34	0.20
BMI	20.19 (3.19)	19.02 (2.63)	1.26	0.22
QIDS	5.51 (4.69)	7.25 (5.51)	−0.98	0.34
SIGH-A	8.84 (7.12)	11.08 (8.99)	−0.79	0.44
Y-BOCS	12.11 (8.11)	17.42 (9.34)	−1.76	0.10
**EAT**	**23.97 (16.88)**	**41.00 (15.50)**	−**3.23**	**0.004**
**EAT-D**	**13.27 (9.80)**	**22.92 (8.74)**	−**3.22**	**0.004**
**EAT-B**	**5.60 (4.67)**	**8.59 (3.42)**	−**2.39**	**0.025**
EAT-O	4.30 (4.62)	8.20 (5.83)	−2.10	0.05
BSQ	59.60 (18.18)	68.75 (17.11)	−1.58	0.13

*BMI, Body Mass Index; BSQ, Body Shape Questionnaire; QIDS, Quick Inventory of Depression; SIGH-A, Structured Interview Guide for the Hamilton Anxiety Index; Y-BOCS, Young-Brown Obsessive-Compulsive Symptoms; EAT, Eating Attitude Test; EAT-D, EAT Diet; EAT-B, EAT Bulimia; EAT-O, EAT Oral*.

*Significant p-values are in red, and the different scores and their T-values are bolded*.

### Neural differences based on task conditions

**Figure 2:**
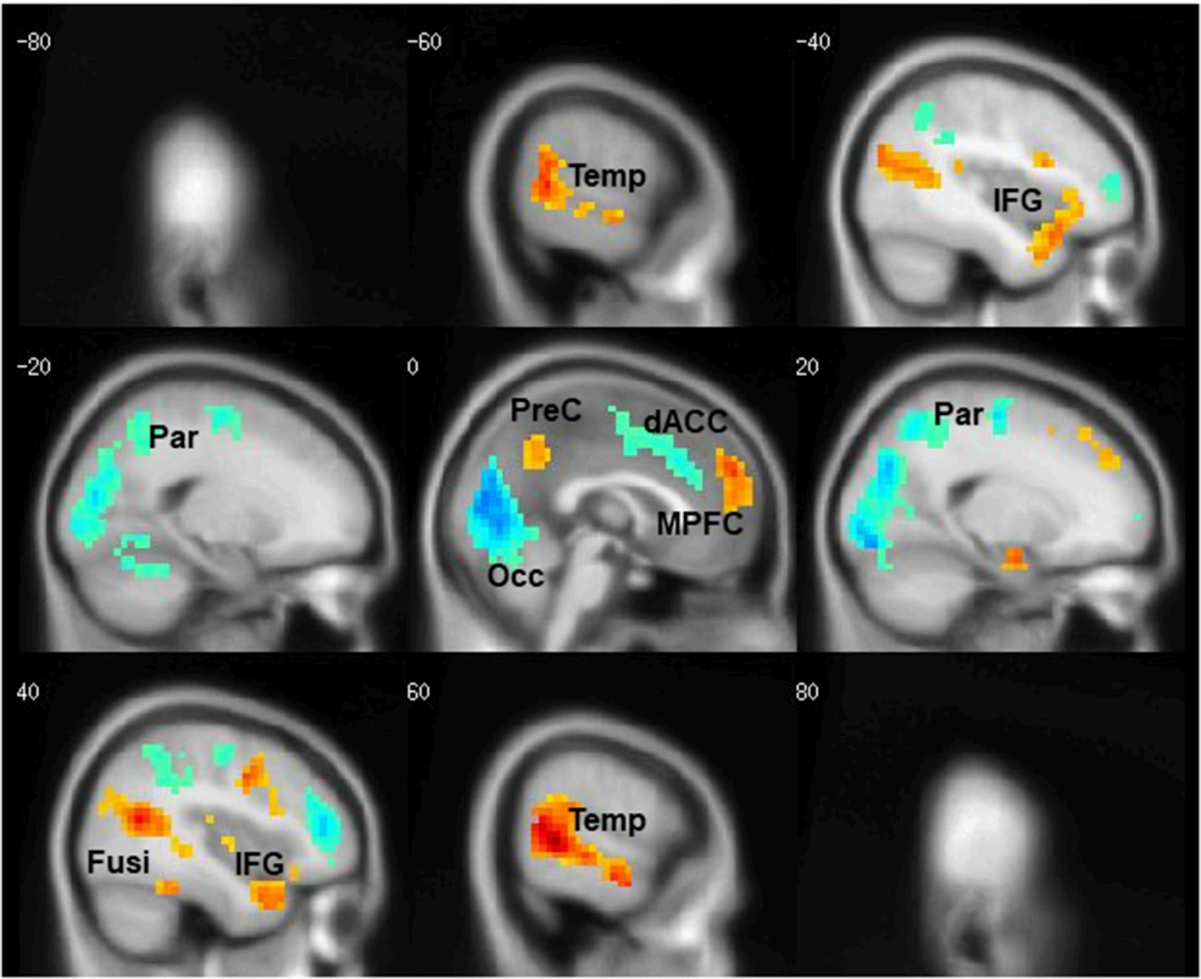
Cortical regions differentially activated by task condition. Areas more active during the People condition are shown in red, areas more active during the Bumper condition are shown in blue.

The task contrasts: People—Bumper and Bumper—People, led to activation of distinct neural circuitry related to social processing and visuospatial analysis (Figure 2). Six regions were activated by the People—Bumper contrast (red) and included the bilateral fusiform gyri, bilateral middle temporal gyrus and inferior frontal gyri, the precuneus, and medial prefrontal cortex. Five regions were activated by the Bumper—People contrast (blue) and included bilateral parietal regions, bilateral middle frontal gyrus, the middle cingulate extending into the dorsal anterior cingulate, and the occipital lobe. Similar results related to activations in this task were previously published, data related to 14 of the participants reported here (McAdams and Krawczyk, 2011). Here, we include additional data from 35 women with AN from the next protocol; this data has not previously been published.

### Neural activation and connectivity differences for rs2254298

**Figure 3:**
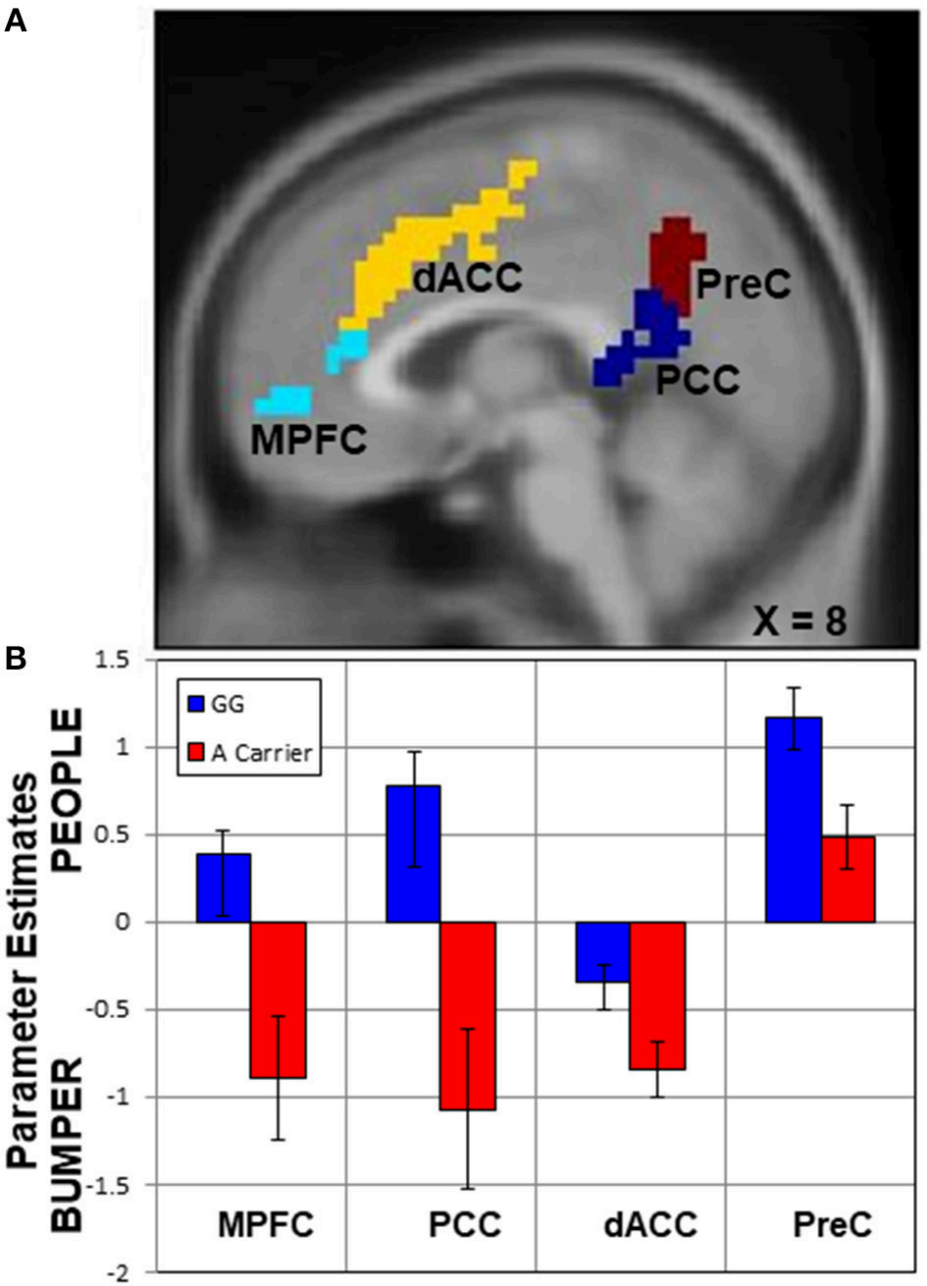
Upper panel **(A)**, regions with activation differences based on SNP rs2254298. Two neural regions emerged from the whole-brain two-sample *t*-test comparing rs2254298GG subjects to rs2254298A subjects, the medial prefrontal cortex (MPFC, cyan) and the posterior cingulate cortex (PCC, blue). Two additional regions modulated by the task conditions also showed differences based on this SNP, the dorsal anterior cingulate (dACC, yellow) and the precuneus (PreC, maroon). In the lower panel **(B)**, means and standard errors of the parameter estimates for each region were extracted for each subject and averaged across each group. In all regions, the rs2254298A carriers have less modulation for the People relative to Bumper condition than the rs2254298GG subjects.

First, in the whole-brain comparison for group differences based on rs2254298, a region of the posterior cingulate cortex extending into the precuneus and a region in medial prefrontal cortex were modulated differently based on SNP for rs2254298 in the People—Bumper contrast (Table 2, Figure 3). Extraction of the β values from this cluster indicated reductions in activation during the People—Bumper contrast for A carriers compared to the GG carriers.

**Table 2:** Whole-brain two-sample *t*-test regions related to fMRI task effects and genotype for rs2254298.

**Condition and Region**	**Neural ROI Characteristics**							**Group Comparisons**^**a**^				
	**Volume (mm^3^)**	**Cluster size**	**Cluster pFWE**	**Peak Z**	**MNI Coordinates**			**GG**	**GA/AA**	***T***	***p***	**Cohen's *d***
					**x**	**y**	**z**					
**EFFECT OF CONDITION: PEOPLE–BUMPER**												
Right Temporal	61,184	956	0.000	Inf	52	−40	4	1.06 (0.57)	0.95 (0.21)	1.00	0.32	n.s.
Left Temporal	45,312	708	0.000	6.49	−52	−60	8	0.82 (0.55)	0.69 (0.33)	1.00	0.33	n.s.
Medial Prefrontal	18,560	290	0.000	5.48	4	56	28	0.90 (0.78)	0.52 (0.70)	1.61	0.12	n.s.
Precuneus	5,440	85	0.001	4.99	−4	−52	40	**1.16 (1.11)**	**0.48 (0.66)**	**2.60**	**0.02**	**0.74**
Right Fusiform	3,968	62	0.008	4.73	32	−32	−20	0.85 (0.86)	0.49 (0.66)	1.50	0.15	n.s.
Left Fusiform	3,584	56	0.013	4.65	−36	−44	−20	0.65 (0.56)	0.36 (0.53)	1.65	0.11	n.s.
**EFFECT OF CONDITION: BUMPER–PEOPLE**												
Occipital	90,368	1,412	0.000	7.15	4	−84	12	−0.85 (0.80)	−1.21 (0.60)	1.69	0.11	n.s.
Right Dorsolateral Prefrontal	6,656	104	0.000	5.28	40	44	8	−0.46(0.79)	−0.94 (0.68)	2.01	0.06	n.s.
Dorsal Anterior Cingulate	24,576	384	0.000	4.94	28	−12	48	−**0.37(0.59)**	−**0.85(0.57)**	**2.54**	**0.02**	**0.83**
Left dorsolateral Prefrontal	3,968	62	0.008	4.33	−28	56	0	−0.53(0.88)	−0.78(0.70)	1.00	0.33	n.s.
Right parietal	16,320	255	0.000	4.31	24	−64	56	−0.41 (0.60)	−0.63 (0.58)	1.10	0.28	n.s.
**EFFECT OF GROUP: rs2254298 GG vs. AG/AA**												
Posterior Cingulate	5,120	80	0.002	4.17	8	−44	12	**0.72 (1.25)**	−**1.07 (1.64)**	**3.47**	**0.003**	**1.23**
Medial Prefrontal Cortex	2,944	46	0.035	3.74	8	32	16	**0.33 (0.75)**	−**0.90 (1.29)**	**3.13**	**0.008**	**1.17**

a. *Group comparisons are based on mean(SD) of extracted parameter estimates; shown each group (GG and GA/AA columns). Areas with significant differences are bolded, and significant p values are in red*.

Second, to further examine the impact of this *OXTR* SNP on task-related neural responses, we extracted β values from all of the regions showing task differences (Figure 2), and compared activation within each region based on the rs2254298 genotype (Table 2). One region from the People—Bumper contrast, the precuneus, showed increased activity in the GG subjects relative to the A carriers and one region from the Bumper—People contrast, the dorsal anterior cingulate, was more active for Bumper stimuli than People stimuli for the A carriers relative to the GG subjects (Table 2, Figure 3).

**Figure 4:**
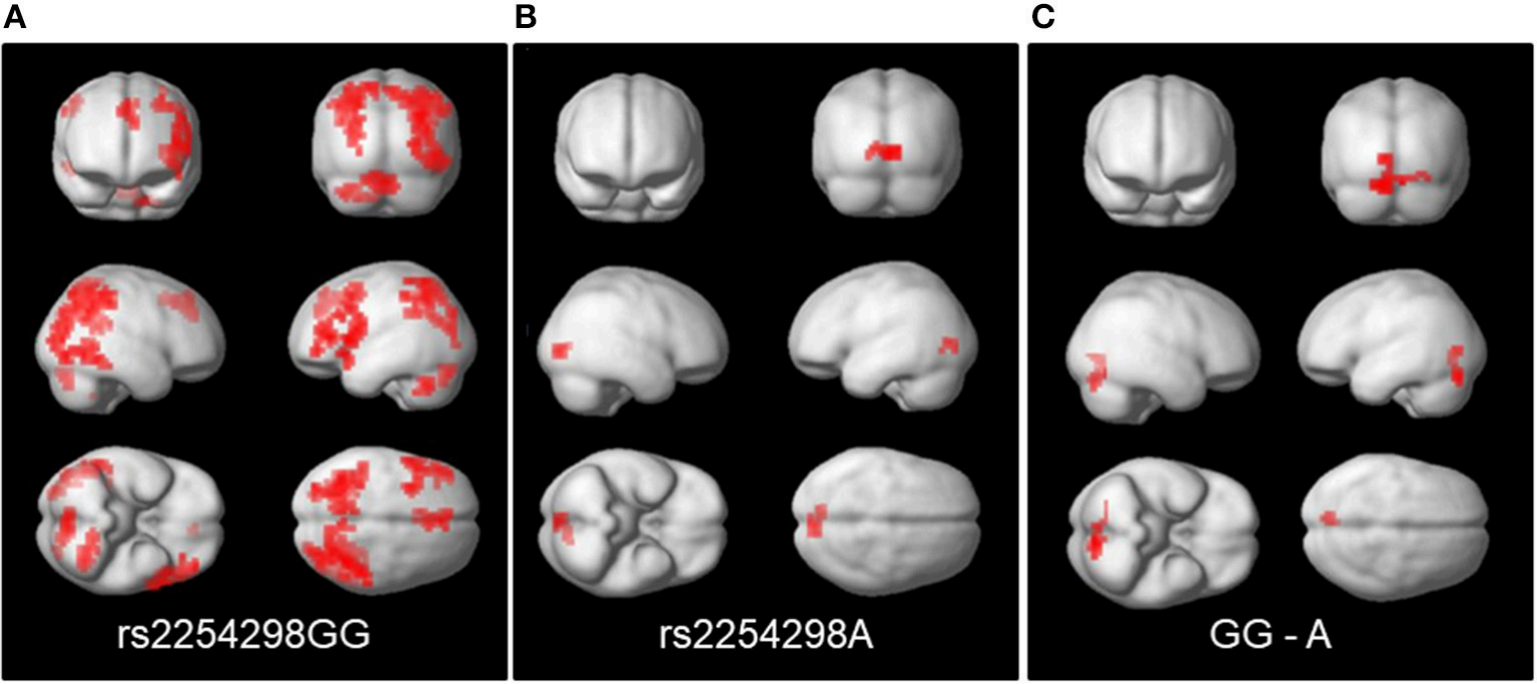
Regions showing connectivity to the posterior cingulate cortex seed (MNI −8, −56, 26). On the left panel **(A)**, the GG subjects showed positive connectivity between the posterior cingulate cortex and regions in bilateral parietal, lingual, cerebellar, and the left middle frontal gyrus and dorsal anterior cingulate. In the center **(B)**, the A subjects showed negative connectivity with the lingual lobe. On the right **(C)**, the comparison of the connectivity in the GG-A subjects resulted in a single cluster in the cerebellum and lingual lobe.

Third, in the People—Bumper task contrast, the rs2254298A and rs2254298GG groups showed different patterns of connectivity for the posterior cingulate cortex seed. Positive connectivity refers to regions that show a positive correlation in their activation patterns whereas negative connectivity refers to areas that are negatively correlated in their activation patterns. Within group, the rs2254298GG subjects showed positive connectivity between the posterior cingulate cortex seed and bilateral parietal regions, the dorsal anterior cingulate, the middle frontal gyrus, the cerebellum, and the lingual lobe, whereas the rs2254298A subjects showed only negative connectivity between the posterior cingulate cortex seed and the lingual lobe (Supplemental Table 3, Figure 4). In the group comparisons, the rs2254298A group showed greater negative connectivity between the posterior cingulate cortex and the occipital lobe and the cerebellum (Occipital, 51 voxels, 3,264 mm^3^, MNI −8, −80, −20) relative to the rs2254298GG group (Figure 4). Although there were no statistically significant group differences using the medial prefrontal cortex seed, the rs2254298A carriers showed positive connectivity between this region and the left middle frontal gyrus, and the rs2254298GG carriers showed negative connectivity between the middle temporal gyri, the precuneus, medial frontal gyrus, and the right superior frontal gyrus (Supplemental Table 3).

### Neural differences based on rs53576

No significantly activated clusters were identified in whole-brain comparisons for A carriers vs. GG subjects based on *OXTR* SNP rs53576. Similarly, there were no significant activation differences in the task-condition ROI analysis using extracted beta values based on being an A carrier or GG subject for SNP rs53576 (Supplemental Table 4).

### Body mass index and genotype

The participants included both currently underweight and weight-restored individuals with AN. To explore the effects of weight-restoration on social brain function within AN, we conducted two analyses. First, we conducted a full factorial whole-brain analysis examining weight-restoration and carrier status for rs2254298. No differences in neural activations were associated with weight and there were no interactions between weight-recovery status and genotype. Second, we examined whether the parameter estimates, beta values, from each of the four ROIs that differed based on the rs2254298 SNP, showed any significant differences related to weight-recovery using ANOVA (Supplemental Table 5). Three of the regions (precuneus, posterior cingulate cortex, and medial prefrontal cortex) showed effects only for rs2254298 genotype and not for BMI. The fourth region, the dorsal anterior cingulate, was modulated both by BMI [F_(1, 45)_ = 6.31, *p* = 0.02] and genotype [F_(1, 45)_ = 6.88, df = 1, *p* = 0.01], with no interaction between the two factors [F_(1, 45)_ = 1.33, *p* = 0.26] (Supplemental Table 5, Supplemental Figure 1).

### Clinical symptoms and regions of interest

Multivariate linear regression was used to evaluate whether any of the office-based clinical assessments for depression (QIDS), anxiety (SIGH-A), eating attitudes (EAT), obsessive-compulsive traits (YBOCS), or body shape (BSQ) correlated with neural activations within the four identified ROIs. None of these clinical symptom measures were significantly related to activations in these ROIs.

## Discussion

This pilot study to explore whether differences in SNPs of *OXTR* in AN could be related to differences in neural activations to social stimuli was supported for SNP rs2254298, but not SNP rs53576. Neural responses to social stimuli in the medial prefrontal cortex, the dorsal anterior cingulate, the posterior cingulate cortex, and the precuneus were reduced in rs2254298A relative to rs2254298GG. These exploratory data were obtained from a small sample in a psychiatric patient population, and limited by unequal group sizes. Nevertheless, AN is ideally suited to examine gene-environment interactions. Specifically, all women with AN have undergone physiological starvation, a clear environmental stressor that may have different effects on people based on genotype. Thus, AN will include some differences in neural functions that are caused by differences in how specific genotypes respond to starvation. Future work to assess the role of these neural pathways and genetic polymorphisms in the context of other psychiatric pathology or examination in less extreme cases of dieting and weight loss will be important to further understand how oxytocin pathways affect human social perception.

The medial prefrontal cortex, posterior cingulate cortex, and precuneus are prominent components of the default mode network (Li et al., 2014) whereas the dorsal anterior cingulate is part of the salience network (Downar et al., 2016). Oxytocin has been proposed to exert its effects via alterations related to salience and motivation toward social stimuli (Love, 2014). Additionally, the posterior cingulate cortex and the occipital lobe showed connectivity differences for the rs2254298A and rs2254298GG groups. In sum, these data suggest that *OXTR* polymorphism differences may subserve some of the neural differences in social perception in AN, and leads to a hypothesis that oxytocin pathways may impact social behavior by coordinating changes in the default mode network when processing social stimuli. Although this is a small sample, the effect-sizes of all results were moderate to large, suggesting that future work examining *OXTR* polymorphisms in the brain should include the examination of midline cortical structures.

Oxytocin systems have been considered in multiple studies as a potential contributor to psychopathology in AN (Table 3). Underweight individuals with AN have lower oxytocin in cerebrospinal fluid levels than controls (Frank et al., 2000). Individuals with AN have lower plasma oxytocin than healthy controls (Monteleone et al., 2016). Overnight oxytocin secretion is reduced acutely in AN (Lawson et al., 2011). Postprandial secretion of oxytocin is decreased in weight-recovered women with AN, while it is increased in individuals with current AN (Lawson et al., 2013). Abnormalities in oxytocin secretion have also been associated with increased severity of disordered eating psychopathology and activation of neural pathways related to food motivation (Lawson et al., 2012). Using a larger sample which included the participants examined here, we previously found that individuals who were A carriers for these two common *OXTR* SNPs (rs53576 and rs2254298) had elevated severity for eating disorder symptoms (e.g., oral control, eating obsessions, and appearance concerns) if they developed AN; however, this was not the case for individuals with bulimia nervosa (Acevedo et al., 2015). Two studies have found that the *OXTR* is more highly methylated in individuals with AN than controls (Kim et al., 2014a; Booij et al., 2015), with one study showing that *OXTR* methylation level was inversely associated with BMI (Kim et al., 2014a). Oxytocin-releasing stimuli cause less increase in oxytocin in individuals with AN than individuals with BN and HC, but this response is normalized after full-weight restoration (Chiodera et al., 1991). Recently, oxytocin administration has been explored as a potential adjunct treatment for AN. In one proof of concept study, intranasal oxytocin administration resulted in significant reductions in attentional biases toward eating-related stimuli and toward fat body parts in individuals with AN, but did not influence juice consumption (Kim et al., 2014b). Here, we found that Eating Attitudes Test scores were significantly higher for rs2254298A carriers compared to rs2244298GG carriers.

**Table 3:** Studies examining oxytocin in anorexia nervosa.

**References**	**N**	**Study purpose and methods**	**Results and conclusions**
Acevedo et al., 2015	AN = 36 AN-WR = 26 BN = 27 HC = 35	Examined whether SNPs of the oxytocin receptor gene correlated with AN clinical symptoms	AN-C and rAN with two common SNPs of the OXTR gene (53576 and 2254298) had elevated eating disorder clinical symptom severity
Chiodera et al., 1991	AN = 7 BN = 8 HC = 9	Measured plasma oxytocin response to two known oxytocin-releasing stimuli (insulin and estrogen)	Oxytocin concentrations increased more in individuals with BN and HC than individuals with AN; after weight restoration, the oxytocin response in AN individuals normalized
Demitrack et al., 1990	AN = 5 BN = 47 HC = 11	Measures cerebrospinal fluid of women with AN, BN, and HC	Restricting AN (but not underweight, normal weight BN, nor normal-weight women) had lower oxytocin cerebrospinal fluid levels
Fetissov et al., 2005	AN = 12 BN = 42 HC = 41	Identified autoantibodies reacting with oxytocin; assessed whether autoantibodies correlated with psychological symptoms	Autoantibodies for oxytocin were higher in AN than BN or controls; bulimia score of EDI-2 correlated with oxytocin levels in both AN and BN
Frank et al., 2000	AN-WR = 10 rBN = 23 HC = 17	Compared cerebrospinal fluid oxytocin in individuals who were weight-recovered from AN-BP, recovered from BN, and HC	Found no significant differences in oxytocin levels in individuals recovered from AN-BP vs. HC and rBN
Hoffman et al., 2012	AN = 20	Compared oxytocin concentration in blood, saliva, and urine samples in women with AN	Plasma oxytocin and salivary oxytocin concentrations were positively correlated, with lower correlations in individuals with self-induced vomiting
Kim et al., 2014b	AN = 31 HC = 33	Examined intranasal oxytocin (vs. placebo) on attention processing for food, shape, and weight stimuli in AN and HC and also on juice consumption by AN and HC	AN vs. HC showed less attentional biases for both food and weight stimuli with oxytocin; oxytocin did not effect juice consumption in either group
Kim et al., 2014c	AN = 31 HC = 33	Examined intranasal oxytocin (vs. placebo) on attention processing for emotional face stimuli (disgust, anger, happy, neutral) in AN and HC.	Oxytocin reduced attentional bias for disgust in both AN and HC. Differences in oxytocin effects for anger varied by group, with AN showing increased vigilance but HC showed decreased vigilance.
Kim et al., 2014a	AN = 15 HC = 36	Examined OXTR methylation and illness severity	OXTR is more highly methylated in individuals with AN than HC; methylation inversely associated with BMI
Kim et al., 2015	AN = 35 BN = 34 HC = 33	Same as 2014c, with BN added. Food intake for 24 h post oxytocin administration measured for all groups.	Oxytocin increased emotion recognition in BN and HC but not AN; decreased calories consumed in BN; no effect in HC or AN
Lawson et al., 2011	AN = 17 HC = 19	Examined the relationship between oxytocin levels, body composition, and bone mineral density in individuals with AN	Subjects with AN (vs. HC) had lower overnight oxytocin secretion; low oxytocin levels were associated with decreased bone mineral density, fat mass, and leptin levels
Lawson et al., 2012	AN = 13 AN-WR = 9 HC = 13	Examined the relationship between abnormal oxytocin secretion in AN and anxiety and depression symptoms	Postprandial secretion of oxytocin after eating was increased in AN (vs. HC) and decreased in wrAN (vs. HC); oxytocin secretion is associated with anxiety and depression symptoms in AN and wrAN
Lawson et al., 2012	AN = 13 AN-WR = 9 HC = 13	Examined the relationship between abnormal oxytocin secretion in AN and eating disorder symptoms and brain activations in a priori regions.	Larger changes in oxytocin in response to feeding was associated with more eating disorder symptomatology and accounted for some of the differences observed in the brain in a priori regions (insula, amygdala, hypothalamus, orbitofrontal cortex) between AN and HC groups.
Monteleone et al., 2016	AN = 23 BN = 27 HC = 19	Compared oxytocin secretion in AN, BN, and HC and relationship between oxytocin and personality traits	AN had lower plasma oxytocin than HC; no difference in plasma oxytocin levels for AN-BP and AN-R; no relationship to oxytocin and personality in AN

*AN, anorexia nervosa; WR, weight-recovered; BP, binge-purge subtype; R, restricting subtype; BN, bulimia nervosa; rBN, recovered bulimia nervosa; HC, healthy controls; EDI-2, Eating Disorder-Inventory-two; OXTR, oxytocin receptor; SNP, single nucleotide polymorphisms; BMI, body mass index*.

Three neuroimaging studies conducted in healthy participants have reported differences in brain volumes for SNP rs2254298. Inoue (Inoue et al., 2010) reported increased amygdala volume in rs2254298A carriers relative to rs2254298GG subjects. Furman (Furman et al., 2011) found increased amygdala volume and decreased total gray matter for rs2254298A carriers. Tost (Tost et al., 2011) found increased gray matter volume in the cingulate and hypothalamus for rs2254298GG subjects, with a more pronounced effect in males. One study recently reported increased activation of the amygdala in response to face stimuli for rs2254298A carriers, which was increased for those participants with early life stress (Marusak et al., 2015). The functional data reported here, reduced modulation within midline cortical structures in response to social stimuli for women with AN that have SNP rs2254298A, extends these studies by providing a deeper understanding of the mechanistic circuitry through which *OXTR* polymorphisms may modulate cognitive and affective processing.

We propose future work to test a model in which rs2254298A status may lead to heightened reactivity of the amygdala for social stimuli (a genotype trait) which may then impact the long-term activation of regions in both the default mode and salience networks for processing social stimuli in women with AN. Both state (such as starvation-related) and trait (predisposition to this illness) abnormalities may thus impact oxytocin function in individuals with AN. Although we considered whether current body mass index might be related to the genotype and neural differences, these studies were limited by the fact that all participants for the imaging studies were required at baseline to be at a stable or increasing weight. Second, as only 12 subjects carried the A allele of rs2254298, the sample is underpowered to identify interactions related to both genotype and weight, and is a pilot project. Similarly, although these participants were in studies designed to compare neural activations in AN to healthy comparison women, only twenty healthy participants volunteered for both the neuroimaging and genetic studies, and only five were A carriers for rs2254298A; therefore we could not determine if this SNP alters functional neural activations to social stimuli in healthy women. Thus, although the proportion of both cohorts (AN and HC) with the A allele of rs2254298 were similar, its effects on social brain function in the healthy women could not be assessed, and remains an important area for future research. In addition, longitudinal studies examining more women with AN at different stages of weight-recovery, may lead to a better understanding of how genotype and starvation contribute to the differences in social function in AN. Future work should consider examining how oxytocin relates to engagement of the default mode network during social behaviors in a wider range of psychopathological samples.

## Ethics statement

The three protocols utilized to collect participant data were approved by the University of Texas at Southwestern Medical Center Institutional Review Board. All participants signed a written informed consent to participate in the study.

## Author contributions

CM and DK designed the study. SA and CM collected the data. MS, KH, SA, and CM processed and analyzed data. All authors contributed to writing and editing the manuscript.

### Conflict of interest statement

The authors declare that the research was conducted in the absence of any commercial or financial relationships that could be construed as a potential conflict of interest.
