# Supplementary material for: Oxytocin Receptor Polymorphism Decreases Midline Neural Activations to Social Stimuli in Anorexia Nervosa
Source: Front Psychol. 2018 Nov 13;9:2183. doi: 10.3389/fpsyg.2018.02183 (PMC6277875; doi:10.3389/fpsyg.2018.02183)

Supplemental Table 1. Demographic and clinical characteristics for rs53576.

|  | rs53576 *(M, SD)* | | rs53576 GG vs. A carrier | |
| --- | --- | --- | --- | --- |
|  | GG (*n* = 22) | GA/AA (*n* = 27) | T | *p* |
| Age | 26.96 (6.92) | 27.15 (9.04) | -.09 | .93 |
| BMI | 19.36 (2.80) | 20.34 (3.28) | -1.12 | .27 |
| QIDS | 5.23 (4.86) | 6.52 (4.96) | .92 | .36 |
| SIGH-A | 8.27 (7.65) | 10.30 (7.55) | -.93 | .36 |
| Y-BOCS | 13.68 (10.29) | 13.23 (7.19) | .17 | .86 |
| EAT | 27.55 (19.86) | 28.63 (16.67) | -.20 | .84 |
| EAT-D | 14.77 (10.83) | 16.33 (10.09) | -.52 | .61 |
| EAT-B | 6.64 (4.96) | 6.07 (4.27) | .42 | .68 |
| EAT-O | 4.41 (5.25) | 5.93 (5.08) | -1.02 | .31 |
| BSQ | 57.46 (18.95) | 65.41 (17.06) | -1.53 | .13 |

*Note:* BMI *=* Body Mass Index; BSQ = Body Shape Questionnaire; QIDS = Quick Inventory of Depression; SIGH-A = Structured Interview Guide for the Hamilton Anxiety Index; Y-BOCS = Young-Brown Obsessive-Compulsive Symptoms; EAT = Eating Attitude Test; EAT-D = EAT Diet; EAT-B = EAT Bulimia; EAT-O = EAT Oral.

Supplemental Table 2. Behavioral data from social attribution task.

|  | Bumper Condition | | | People Condition | | |
| --- | --- | --- | --- | --- | --- | --- |
| rs53576 | GG | GA/AA | P | GG | GA/AA | P |
| Reaction Time^1^ (ms) | 1251(477) | 1093(374) | 0.13 | 1172(373) | 1029(338) | 0.13 |
| Percent Correct | 56(18) | 58(15) | 0.11 | 81(12) | 75(12) | 0.12 |
| rs2254298 |  |  |  |  |  |  |
| Reaction Time^1^ (ms) | 1194 (455) | 1287(826) | 0.69 | 1055(341) | 1336(673) | 0.16 |
| Percent Correct | 57(17) | 48(17) | 0.09 | 78(12) | 79(11) | 0.88 |

^1^Reaction Time is in milliseconds.

Supplemental Table 3. Within-group connectivity clusters observed for the posterior cingulate cortex (PCC) and medial prefrontal cortex (MPFC) seeds for rs2254298. Missing comparisons did not show any significant clusters (cluster P_FWE_ < 0.05; voxel P < 0.005). Both positive and negative connectivity were examined for the GG and A subjects separately.

| Region | Neural ROI Characteristics | | | | | |
| --- | --- | --- | --- | --- | --- | --- |
|  | Volume  (mm^3^) | Cluster  Size | Peak  Z | MNI Coordinates | | |
|  |  |  |  | x | y | z |
| PC SEED AT MNI -8, -56, 26, GG SUBJECTS ONLY, POSITIVE CONNECTIVITY | | | | | | |
| Right Parietal | 26,112 | 408 | 5.06 | 28 | -60 | 28 |
| Left Parietal | 14,272 | 223 | 4.41 | -36 | -48 | 32 |
| Left Middle Frontal Gyrus | 10,304 | 161 | 4.18 | -28 | 8 | 44 |
| Dorsal Anterior Cingulate | 3,456 | 54 | 4.04 | -4 | 44 | 32 |
| Cerebellum | 3,776 | 59 | 3.90 | -4 | -80 | -28 |
| Left Lingual/Cerebellum | 3,200 | 50 | 3.50 | -12 | -52 | -44 |
| PC SEED AT MNI -8, -56, 26, A SUBJECTS ONLY, NEGATIVE CONNECTIVITY | | | | | | |
| Right Lingual | 2,112 | 33 | 3.74 | 8 | -84 | 0 |
| MPFC SEED at MNI -6, 52, -2, A SUBJECTS ONLY, POSITIVE CONNECTIVITY | | | | | | |
| Left Middle Frontal Gyrus | 2,496 | 39 | 3.84 | -36 | 44 | 12 |
| MPFC SEED AT MNI -6, 52, -2, GG SUBJECTS ONLY, NEGATIVE CONNECTIVITY | | | | | | |
| Right Middle Temporal Gyrus | 23,680 | 370 | 4.95 | 48 | 0 | -24 |
| Left Middle Temporal Gyrus | 6,784 | 106 | 4.60 | -44 | -76 | 28 |
| Left Lingual/Cerebellum | 2,880 | 45 | 4.45 | -20 | -80 | -20 |
| Right Superior Frontal | 2,624 | 41 | 4.32 | 20 | 44 | 40 |
| Precuneus | 13,632 | 213 | 4.20 | 12 | -52 | 44 |
| Medial Frontal Gyrus | 3,520 | 55 | 4.08 | 4 | 60 | 12 |

Supplemental Table 4. Whole-brain two-sample t-test regions related to fMRI task effects and genotype for rs53576.^a^

| Condition and Region | Neural ROI Characteristics | | | | | | Group Comparisons for rs53576 | | | | | |
| --- | --- | --- | --- | --- | --- | --- | --- | --- | --- | --- | --- | --- |
|  | Volume  (mm^3^) | Cluster  Size | Peak  Z | MNI Coordinates | | | GG | GA/AA | T | *p* | Effect Size |  |
|  |  |  |  | x | y | z |  |  |  |  |  |  |
| Effect of Condition: People – Bumper | | | | | | | | | | | |  |
| Right Temporal | 61,184 | 956 | Inf | 52 | -40 | 4 | 1.05 (0.44) | 1.02 (0.56) | 0.21 | 0.83 | n.s. |  |
| Left Temporal | 45,312 | 708 | 6.49 | -52 | -60 | 8 | 0.75 (0.45) | 0.83 (0.55) | -0.55 | 0.59 | n.s. |  |
| Medial Prefrontal | 18,560 | 290 | 5.48 | 4 | 56 | 28 | 0.81 (0.81) | 0.81 (0.77) | 0.01 | 0.99 | n.s. |  |
| Precuneus | 5,440 | 85 | 4.99 | -4 | -52 | 40 | 1.11 (1.07) | 0.89 (1.05) | 0.74 | 0.47 | n.s. |  |
| Right Fusiform | 3,968 | 62 | 4.73 | 32 | -32 | -20 | 0.78 (0.64) | 0.75 (0.95) | 0.16 | 0.88 | n.s. |  |
| Left Fusiform | 3,584 | 56 | 4.65 | -36 | -44 | -20 | 0.53 (0.59) | 0.62 (0.54) | -0.56 | 0.58 | n.s. |  |
| Effect of Condition: Bumper – People | | | | | | | | | | | |  |
| Occipital | 90,368 | 1,412 | 7.15 | 4 | -84 | 12 | -0.93 (0.58) | -0.94 (0.90) | 0.06 | 0.95 | n.s. |  |
| Right Dorsolateral Prefrontal | 6,656 | 104 | 5.28 | 40 | 44 | 8 | -0.54 (0.72) | -0.61 (0.85) | 0.29 | 0.77 | n.s. |  |
| Dorsal Anterior Cingulate | 24,576 | 384 | 4.94 | 28 | -12 | 48 | -0.55 (0.63) | -0.43 (0.60) | -0.69 | 0.49 | n.s. |  |
| Left dorsolateral Prefrontal | 3,968 | 62 | 4.33 | -28 | 56 | 0 | -0.81 (0.85) | -0.42 (0.81) | -1.64 | 0.11 | n.s. |  |
| Right parietal | 16,320 | 255 | 4.31 | 24 | -64 | 56 | -0.38 (0.47) | -0.54 (0.68) | 0.94 | 0.35 | n.s. |  |
| Effect of Group Clusters from the Whole-Brain Two-Sample T-test for rs2254298 A carrier vs GG^b^ | | | | | | | | | | | |  |
| Posterior Cingulate | 5,120 | 80 | 4.17 | 8 | -44 | 12 | 0.36 (1.70) | 0.21 (1.43) | 0.33 | 0.75 | n.s. |  |
| Medial Prefrontal Cortex | 2,944 | 46 | 3.74 | 8 | 32 | 16 | -0.07 (1.04) | 0.12 (1.05) | -0.63 | 0.53 | n.s. |  |

1. There were no whole-brain clusters related to SNP rs53576 A carriers vs GG, so means and SD related to having the SNP rs53576 A carriers vs GG were considered for the two clusters obtained from the whole-brain two-sample t-test comparing rs2254298 A carrier to GG, as well as the clusters related to the effects of condition.

Supplemental Table 5. Effects of weight and genotype for the four clusters with genotype effects.

| Effect | Df | F | p |
| --- | --- | --- | --- |
| Precuneus from effect of condition (People – Bumper) | | | |
| BMI | 1 | 1.32 | 0.26 |
| **rs2254298** | **1** | **4.02** | **0.05** |
| BMI x rs2254298 | 1 | 0.72 | 0.41 |
| Posterior Cingulate from effect of group (rs2254298) | | | |
| BMI | 1 | 0.03 | 0.87 |
| rs2254298 | 1 | 15.41 | **<0.001** |
| BMI x rs2254298 | 1 | 0.40 | 0.53 |
| Medial Prefrontal Cortex from effect of group (rs2254298) | | | |
| BMI | 1 | 0.17 | 0.68 |
| rs2254298 | 1 | 16.18 | **<0.001** |
| BMI x rs2254298 | 1 | 0.98 | 0.33 |
| Dorsal Anterior Cingulate from effect of condition (Bumper – People) | | | |
| BMI | 1 | 6.31 | **0.02** |
| rs2254298 | 1 | 6.88 | **0.01** |
| BMI x rs2254298 | 1 | 1.33 | 0.26 |

*Note:* BMI group compared weight-restored (n = 25, BMI > 19 at scan) to underweight (n = 24, BMI < 19); rs2254298 group compared individuals who are A carriers (n = 12) vs. GG genotype (n = 37).

Supplemental Figure 1. Parameter estimates extracted for groups constructed based on both genotype (rs2254298 A carrier or GG) and BMI (underweight at scan, -U; weight-restored –W). There were 6 rs2254298A carriers in each BMI group, 19 in the GG-W group, and 18 in the GG-U group.


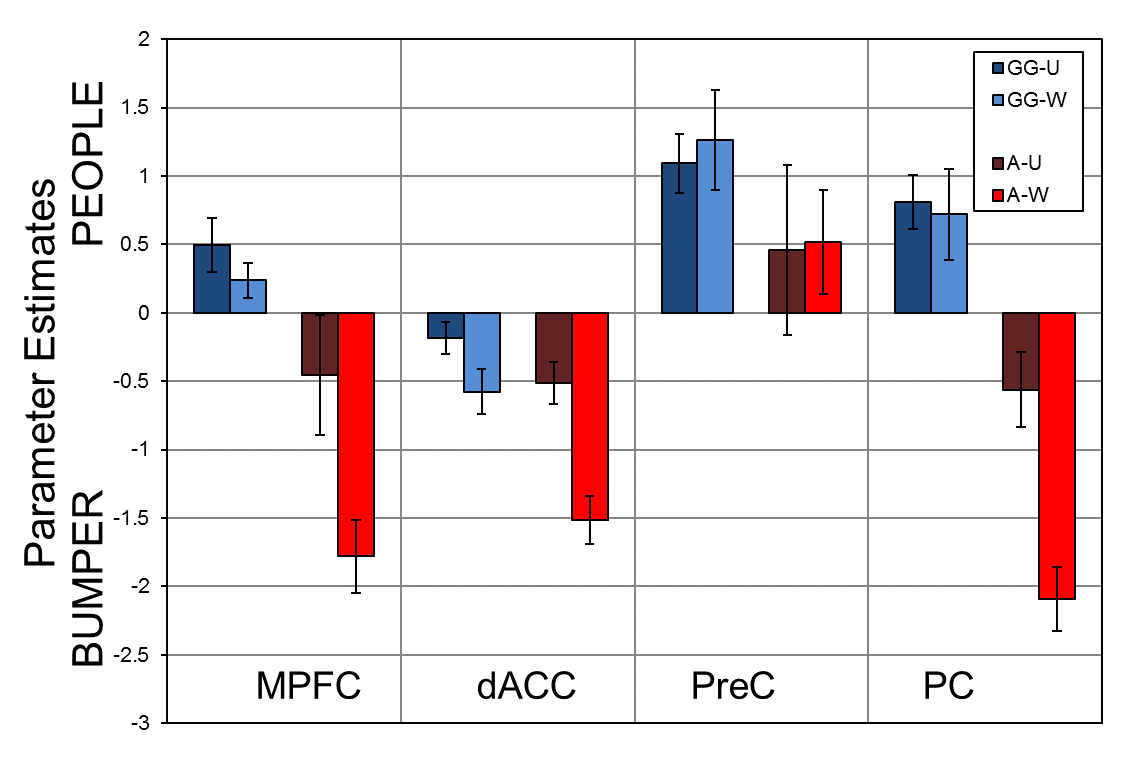

Supplement: Supplementary file 1 [file Table_1.doc]
